# Tailoring the Holocellulose Fiber/Acrylic Resin Composite Interface with Hydrophobic Carboxymethyl Cellulose to Enhance Optical and Mechanical Properties

**DOI:** 10.1021/acs.biomac.4c00295

**Published:** 2024-05-07

**Authors:** Li Zha, Max Yan, Lars A. Berglund, Qi Zhou

**Affiliations:** † Division of Glycoscience, Department of Chemistry, School of Engineering Sciences in Chemistry, Biotechnology and Health, KTH Royal Institute of Technology, AlbaNova University Centre, Stockholm SE-106 91, Sweden; ‡ Department of Applied Physics, School of Engineering Sciences, KTH Royal Institute of Technology, Stockholm SE-114 19, Sweden; § Wallenberg Wood Science Center, Department of Fibre and Polymer Technology, KTH Royal Institute of Technology, Teknikringen 56, Stockholm SE-100 44, Sweden

## Abstract

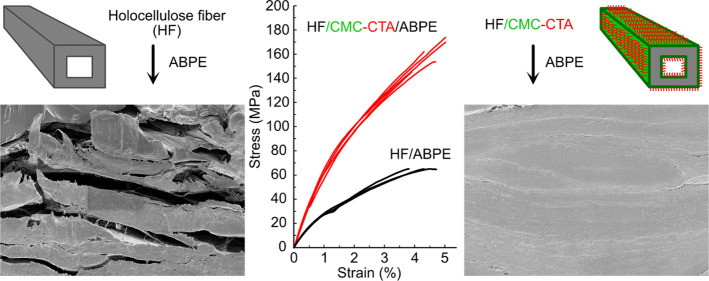

Interface engineering is essential for cellulosic fiber-reinforced polymer composites to achieve high strength and toughness. In this study, carboxymethyl cellulose (CMC) functionalized with hydrophobic quaternary ammonium ions (QAs) were utilized to modify the interface between holocellulose fibers (HF) and acrylic resin. The wet HF/CMC papers were prepared by vacuum filtration, akin to papermaking, followed by cationic ion exchange with different hydrophobic QAs. Subsequently, the modified papers were dried, impregnated with an acrylic resin monomer, and cured to produce transparent composite films. The effect of the hydrophobic QA moieties on the structure and optical and mechanical properties of the HF/CMC/acrylic resin composites were investigated. The composite film with cetyltrimethylammonium (CTA)-functionalized CMC showed high optical transmittance (87%) with low haze (43%), while the composite film with phenyltrimethylammonium (PTMA)-functionalized CMC demonstrated high Young’s modulus of 7.6 GPa and high tensile strength of 180 MPa. These properties are higher than those of the composites prepared through covalent interfacial modification strategies. The results highlighted the crucial role of hydrophobic functionalized CMCs in facilitating homogeneous resin impregnation in the HF fiber network, producing a composite with enhanced interfacial adhesion strength, increased optical transparency, and mechanical strength. This facile use of hydrophobic CMCs as interfacial compatibilizers provides an energy-efficient route for preparing transparent, thin, and flexible composite films favorable in optoelectronic applications.

## 1 Introduction

One of the major obstacles to unleash the full potential of cellulose fiber or nanofiber-reinforced composite materials is the far-from-optimal interfaces, which include not only the interface between cellulose and polymer matrix, but also the interface and bonding between the individual cellulose fibers or nanofibers.^1−3^ The unfavorable fiber–matrix interface originates primarily from the low compatibility between the hydrophilic cellulose and the hydrophobic polymer matrices. This leads to poor distribution of cellulose fibers or nanofibers in the composites as well as low adhesion strength between cellulose and the polymer matrix, creating interfacial defects that compromise optical and mechanical performances of the composite.^4^ Therefore, the hydrophilic character of cellulose should be reduced, often by the surface modification of cellulose to incorporate hydrophobic moieties.

Covalent surface chemical modifications, including acetylation,^5−7^ succination,^8^ and polymer grafting with polycaprolactone (PCL),^9−17^ polyethylene glycol (PEG),^18,19^ and monoepoxy phenyl glycidyl ether (PGE),^20^ have been performed on cellulose fibers and nanofibers to improve the strength of interfacial adhesion between cellulose and hydrophobic polymers. However, covalent surface hydrophobic modifications of cellulose have significant drawbacks as the use of harsh chemicals and tedious organic solvent exchange processes are often inevitable. In addition, chemical modifications, particularly at high degree of substitution, have induced degradation of cellulose fibers, decrease in cellulose crystallinity,^8,21^ and refractive-index mismatching.^5^ To avoid these drawbacks, a promising approach is to use quaternary ammonium ions (QAs) to incorporate hydrophobic moieties onto anionic charged cellulose surfaces through electrostatic interactions. This method was first demonstrated on acid-hydrolyzed cellulose nanocrystals (CNCs) to obtain high surface hydrophobicity.^22,23^ It was also proven effective in modifying 2,2,6,6-tetramethyl-1-piperidinyloxy (TEMPO)-oxidized cellulose nanofibers (CNFs) to increase hydrophobicity^24^ and achieve better dispersion^25^ as well as redispersion^26^ in organic solvent. Particularly, TEMPO-oxidized CNF modified by the QA moieties with more bulky hydrophobic structures showed higher enhancement in hydrophobicity.^24,25^ Hydrogenated acrylonitrile-butadiene rubber reinforced with 5 wt % TEMPO-oxidized CNF bearing different tetraalkylammonium counterions has been prepared. The incorporation of hydrophobic counterions resulted in higher strain to failure as well as lower coefficient of thermal expansion for the composites.^27^ Composites from waterborne polyurethane (PU) and TEMPO-oxidized CNF bearing tetrabutylammonium counterions have also demonstrated strong enhancement in mechanical properties due to homogeneous infiltration and integration of PU resin into the CNF network.^28^

In addition to improving the fiber–matrix interface, fiber–fiber bonding is also important as it is often weakened due to mechanically or chemically damaged fiber or nanofiber surfaces during the preparation and application processes. High content of cellulose nanofibers in the hydrophobic polymer matrices also tends to result in poor nanofiber dispersion and formation of agglomerates, impairing material performance of the composites.^4,29^ To address these issues, resin monomers or prepolymers have been impregnated with a preformed cellulose nanofiber network followed by further polymerization and curing.^30^ Holocellulose fibers obtained from mild peracetic acid (PAA) delignification of wood fibers are promising as the fibers show less surface damage with higher amount of native hemicellulose preserved, as compared with the conventional wood pulp fibers.^2,13,31−33^ Besides CNF nanopaper,^34−37^ bacterial cellulose (BC) pellicles,^5,38−40^ top-down delignified wood structures,^41,42^ holocellulose fiber,^43^ and pulp fiber mats^44^ have also been used as the preformed cellulose networks. Chemical modifications have also been studied on these cellulose networks prior to polymer impregnation. Surface acetylation on BC sheets,^5,40^ top-down delignified wood,^42^ and wood cellulose nanofibers^36,37^ could improve optical transparency of the composites by reducing the formation of optical defects, thus to alleviate light scattering in the composites.

**Figure 1:**
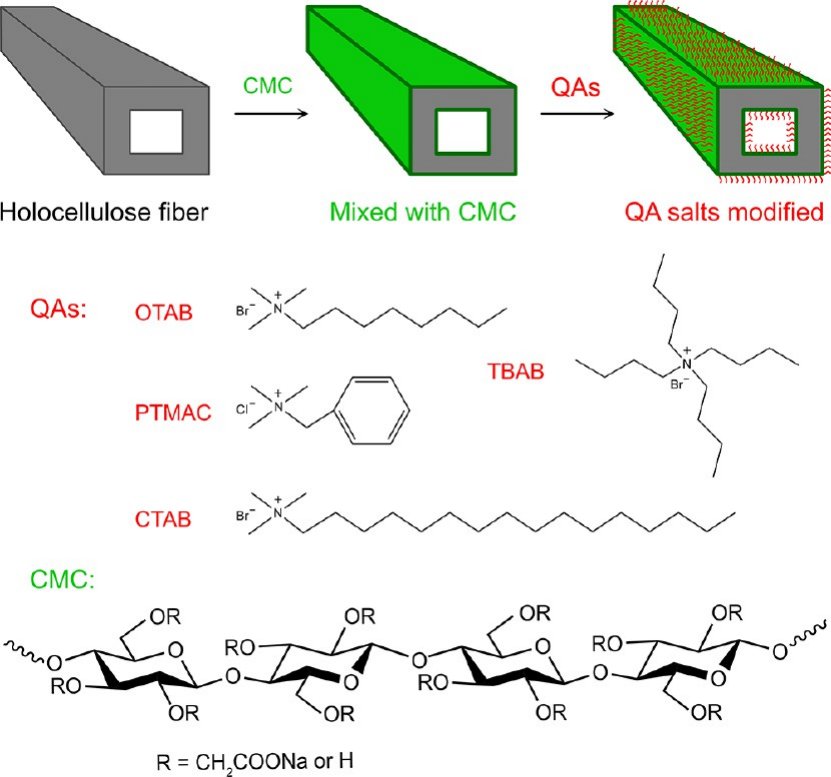
Schematic illustration for modification of holocellulose fiber with CMC functionalized with quaternary ammonium salts.

Carboxymethyl cellulose (CMC), an anionic water-soluble cellulose derivative, has a high affinity with cellulose fibers and is widely used in the paper industry to facilitate stronger fiber-to-fiber bonds for improved paper strength and durability. Adsorption of CMC onto cellulose nanofibers was utilized for nanofiber redispersion^45,46^ and enhancement of nanopaper wet strength.^47^ CMC was also found to interact with hemicellulose and form dense blended films with potential in packaging applications.^48,49^ In this work, we have developed a facile approach for surface hydrophobic modification of holocellulose fibers using CMC functionalized with quaternary ammonium salts (QAs). The holocellulose fibers were mixed with CMC and vacuum filtrated to obtain a wet holocellulose paper, which was further treated with quaternary ammonium salts including cetyltrimethylammonium bromide (CTAB), octyltrimethylammonium bromide (OTAB), tetrabutylammonium bromide (TBAB), and phenyltrimethylammonium chloride (PTMAC), as shown in Figure 1. The hydrophobic moieties were incorporated as counterions onto the CMC, which was already integrated within the holocellulose fibers. These hydrophobic holocellulose fiber/CMC papers were dried and then impregnated with an acrylic resin monomer, a bifunctional acrylate, which is 2,2-bis[4-(acryloxy polyethoxy)phenyl] propane (A-BPE-10), followed by UV curing. The composites were obtained with the hydrophobic functionalized CMCs acting as the compatibilizer at the interface between the holocellulose fibers and acrylic resin. Optical transmittance is sensitive to small defects in the composite arising from incomplete resin impregnation and poor interface bonding. The structures and optical and mechanical properties of the obtained composites with and without the hydrophobic functionalized CMC were characterized and compared to acquire insights into structure-property relationships of these composite materials.

## 2 Experimental Section

### Materials

Holocellulose fibers (HF) were prepared following a reported method using mild PAA delignification on softwood spruce matchsticks.^32^ CMC sodium salt (*M*_*w*_ 250 kDa, DS 0.9, Product No. 419303), CTAB (≥98%, Product No. H5882), OTAB (≥98%, Product No. 75091), TBAB (≥98%, Product No. 426288), and PTMAC (≥98%, Product No. 199168) were purchased from Sigma-Aldrich and used without further purification. UV-curable acrylic resin monomer A-BPE-10 with a refractive index of 1.516 was supplied by Shin-Nakamura Chemical Co., Ltd., Japan, and 2,2 dimethoxy-2-phenylacetophenone (99%, Sigma-Aldrich, Product No. 196118) was used as the photoinitiator.

### Preparation of Hydrophobic HF/CMC Papers

In general, 200 g of HF water suspension (0.1 wt %) was mixed with 3 g of CMC water solution (1 wt %) by magnetic stirring at 23 °C for 24h. The suspension mixture (pH = 6.9) was then homogenized by an Ultra-Turrax disperser (T-25, IKA) at 20 000 rpm for 2 min before vacuum filtration through a Duapore membrane filter (0.22 μm) with a diameter of 90 mm to prepare a wet HF/CMC paper. To ensure the irreversible attachment of CMC on HF,^50^ the wet HF/CMC paper was immersed in 200 mL 10 mM HCl solution for 2 h and then rinsed in 200 mL of deionized water 3 times. Subsequently, ion exchange was performed by soaking the wet paper in 200 mL of QA water solution for 12 h, and the pH was adjusted to 7.5 using 10 mM NaOH. To optimize the ion exchange rate, the effect of molar ratio between the QA and the carboxylate groups in CMC was studied with CTAB. To achieve a molar ratio of 0.5:1.0, 1.2:1.0, 2.0:1.0 between CTAB and the carboxylic acid of the HF/CMC paper, 200 mL 0.25, 0.6, and 1.0 mM CTAB water solutions were applied. After exchanging the counterions, the wet paper was rinsed in deionized water thoroughly to remove the sodium halide salts and dried by using Rapid-Köthen at 93 °C under a reduced pressure of 95 kPa for 12 min. With the incorporation of different QAs as counterions, the HF/CMC-QAs papers were coded as HF/CMC-CTA, HF/CMC-OTA, HF/CMC-TBA, and HF/CMC-PTMA. The neat HF and HF/CMC papers were prepared following the same procedure, including homogenization, filtration, protonation, washing, and drying. The moisture contents in these HF/CMC papers were measured after being stored at a relative humidity (RH) of 50% and 23 °C for 2 days.

### Preparation of HF/Acrylic Resin Composites

The neat HF and HF/CMC, and the hydrophobic HF/CMC-QAs papers were dried in a vacuum oven at 50 °C for 48 h. The dried papers were impregnated with acrylic resin monomer A-BPE-10 containing 1 wt % photoinitiator under a reduced pressure for 12h. The resin-impregnated papers were cured under UV light (365 nm, 230 V-50 Hz, XX-15S UV Bench Lamp, Upland, CA, USA) for 2 h to prepare the HF/ABPE, HF/CMC/ABPE, and HF/CMC-QAs/ABPE composites.

### Characterizations

Attenuated total reflectance Fourier transform infrared (ATR-FTIR) spectra were recorded in ambient conditions on a PerkinElmer Spectrum System 2000 FTIR spectrometer equipped with a MKII Golden Gate, single reflection ATR system from Specac Ltd., London, UK. The measurements were performed over the wavelength range 4000–600 cm^–1^ with a scanning resolution of 4 cm^–1^ and 64 scans. The contact angle measurements were performed on an optical tensiometer (Theta Lite from Biolin Scientific, Finland) with a water droplet of 4 μL. Tensile tests for the HF/CMC-QAs papers and HF/CMC-QAs/ABPE composites were performed on an Instron 5944 single column tester equipped with a 500 N load cell and an advanced video extensometer. The width of the specimen was 3 mm, and the gauge length was 20 mm. The specimens were conditioned at a RH of 50% for 2 days before measurements. The stress–strain curves were recorded at a strain rate of 10% per min at 23 °C and a RH of 50%. For each sample, at least 5 specimens were measured. Total transmittance and haze measurements were carried out under ambient conditions according to ASTM D1003: Standard Test Method for the Haze and Luminous Transmittance of Transparent Plastics. The sample was positioned in front of the input port of an integrating sphere. A supercontinuum white-light source (model SC-5, YSL Photonics) was applied as an incident beam. Transmitted light was captured in the integrating sphere. Through a fiber port on the sphere, the transmitted light is channeled to a spectrometer for spectrum measurement. Each sample (thickness = 60 μm) was measured at 3 different sample positions, and their average gives the final measured spectrum. Transmittance and haze reported are average values in the wavelength range of 500–900 nm. The morphologies of the composite papers and films were studied by field-emission scanning electron microscopy (FE-SEM, Hitachi S-4800) operating at 1 kV. The samples were coated with a thin layer of gold–palladium in a Cressington 208HR sputter coater prior to FE-SEM analysis.

## 3 Results and Discussion

### 3.1 Structure and Properties of Hydrophobic HF/CMC Papers

The HF prepared from spruce wood showed an average diameter of 30 μm and a length of several millimeters (Figure S1). Previously, carboxymethylation with a degree of substitution (DS) of 0.1 on bleached softwood fibers was found sufficient to isolate cellulose nanofibrils.^51^ In addition, a DS of 0.1–0.4 for cellulose esterification was required to achieve topochemical surface modification of cellulose nanofibers.^52^ Therefore, CMC with a *M*_*w*_ of 250 kDa and DS = 0.9 was mixed with the HF at a loading dosage of 150 mg CMC per gram of HF in 0.1 wt % HF water suspension at neutral pH. After vacuum filtration, protonation, washing, and drying, the ratio of CMC and HF in the composite paper was 12.5:100 as measured by weight, corresponding to a DS of about 0.1 for carboxymethyl groups in the HF/CMC paper. Therefore, the carboxylic acid content of the prepared HF/CMC paper was 0.47 mmol/g.

**Figure 2:**
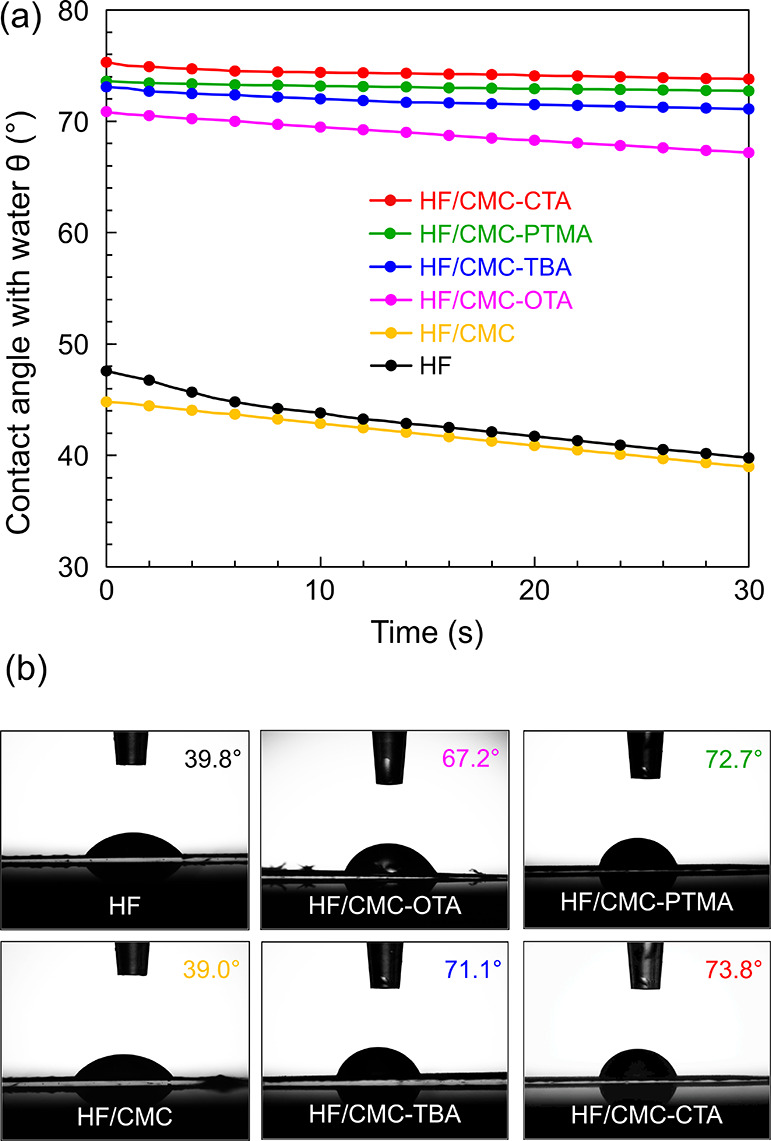
(a) Time-dependent contact angles of water droplets on the surfaces of the HF/CMC-OTA, HF/CMC-TBA, HF/CMC-PTMA, and HF/CMC-CTA papers as compared with those of the neat HF and HF/CMC papers. (b) Images of a water droplet on these paper surfaces after 30 s in contact angle measurements.

The formation of ion-association complexes between CMC and quaternary ammonium cations through electrostatic and hydrophobic interactions have been reported previously.^53,54^ To optimize the hydrophobic modification of the HF/CMC paper using QAs, CTAB water solutions with different concentrations were applied during the cationic ion exchange step. The hydrophobicity of the CTAB-modified HF/CMC papers were evaluated by the contact angle measurements (Figure S2). Based on the amount of carboxylic acid in the HF/CMC paper, 0.5, 1.2, and 2.0 mol equiv of CTAB were added in the water bath. The final associated amounts of CTAB in the HF/CMC papers were 0.42, 0.75, and 0.92 mol equiv, respectively, as measured by weights. The water contact angles of the HF/CMC-CTA papers were 65.4°, 73.8°, and 75.4°, respectively. Considering the incorporation efficiency of CTAB and the hydrophobic performance, the loading amount of 1.2 mol equiv was also used for the other QAs. Correspondingly, the associated amounts of OTA, TBA, and PTMA in the HF/CMC papers were 0.74, 0.75, and 0.77 mol equiv, respectively. Their water contact angles as a function of time were measured and compared with unmodified papers (Figure 2). The neat HF and HF/CMC papers initially showed contact angles of below 47.8° and 45.0°, respectively. After 30 s, the contact angles decreased to 39.8° and 39.0° correspondingly due to water wetting and penetrating on a relatively hydrophilic surface. The counterion exchange with the QAs successfully increased the water contact angle owing to the incorporation of hydrophobic moieties on the CMC, achieving water contact angles of 67.2°, 71.1°, 72.7°, and 73.8° for the HF/CMC-OTA, HF/CMC-TBA, HF/CMC-PTMA, and HF/CMC-CTA papers, respectively. The water penetration in the HF/CMC papers was significantly hindered as the contact angle was kept almost constant after 30 s. Comparing the HF/CMC paper modified by CTA with the one modified by OTA, a longer alkyl chain is preferred to achieve higher surface hydrophobicity. Interestingly, the HF/CMC-TBA and HF/CMC-PTMA papers also showed higher surface hydrophobicity than HF/CMC-OTA, owing to the adequate surface coverage by the bulky quaternary ammonium counterions with branched or cyclic structures. Similar behavior was previously reported for the tetrabutylammonium modified TEMPO-oxidized CNF film, which showed an increase of water contact angle from 60° to 100°.^24^

**Figure 3:**
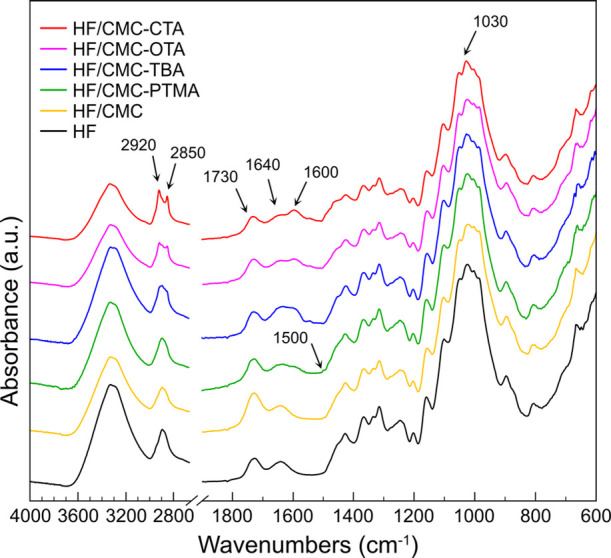
FTIR spectra of the HF/CMC-CTA, HF/CMC-OTA, HF/CMC-TBA, and HF/CMC-PTMA papers as compared with the neat HF and HF/CMC papers.

The successful incorporation of the QAs to the HF/CMC paper was further verified by FTIR analysis, as shown in Figure 3. The neat HF showed a band at 1730 cm^–1^ that corresponds to the C=O stretching vibration from glucuronic acid in the preserved hemicellulose by mild PAA delignification. Hemicellulose is beneficial for the formation of interfibrillar interphase, enhancing optical and mechanical properties of holocellulose-based materials.^31,33^ The peaks at 1640 and 1030 cm^–1^ corresponding to the O–H bending vibration of water sorption and the glucose ring stretching vibration, respectively, were characteristics for cellulose and hemicellulose. With the addition of CMC in HF and protonation with 10 mM HCl, the peak intensity at 1730 cm^–1^ increased in the FTIR spectrum of the HF/CMC paper. After the successful formation of ion-association complexes with the different quaternary ammonium ions, the intensity of the band at 1730 cm^–1^ decreased and a new peak at 1600 cm^–1^ appeared, indicating the formation of carboxylate salt-type structures through electrostatic interactions. Furthermore, the asymmetrical and symmetrical CH_2_ stretches from the long alkyl chain can be observed at 2920 and 2850 cm^–1^, respectively, in the HF/CMC-CTA, HF/CMC-OTA, and HF/CMC-TBA papers. The intensity of these two peaks increased with the increasing alkyl chain length of the counterions in the sequence of TBA, OTA, and CTA. In the spectrum of the HF/CMC-PTMA paper, a small peak at 1500 cm^–1^ emerged, corresponding to the aromatic ring skeleton from the phenyl group.

**Figure 4:**
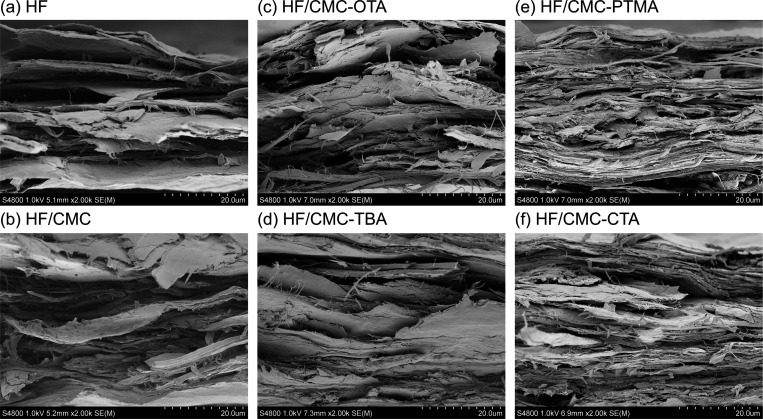
FE-SEM images of tensile fracture surfaces of the HF/CMC-OTA, HF/CMC-TBA, HF/CMC-PTMA, and HF/CMC-CTA papers as compared with the neat HF and HF/CMC papers.

The tensile fractured surface morphology of the hydrophobic HF/CMC papers characterized by FE-SEM are shown in Figure 4 and images of the paper surfaces are shown in Figure S3. The HF paper showed a layered yet porous structure with clearly separated fiber layers on the tensile fractured surface (Figure 4a) and a fiber network structure on the paper surface (Figure S3a). With the addition of CMC, the gap between fiber layers was filled, and a denser fiber network was observed on the surface (Figure S3b). After functionalizing with the QAs, the surfaces of the HF/CMC papers became even denser (Figure S3c–f), while denser layers of fiber network with less fiber pull out were presented at the tensile fracture surfaces, particularly for the HF/CMC-PTMA (Figure 4e) and HF/CMC-CTA papers (Figure 4f). This is consistent with the increase in density and stiffness of the HF paper after the addition of CMC and further functionalization with the QAs (Table S1). In addition, with the incorporation of hydrophobic moieties, the HF/CMC paper became less hygroscopic, indicated by decreased moisture content from 12.5 wt % for the HF/CMC paper to 6.3 wt % for the HF/CMC-CTA paper at RH 50%. A similar phenomenon was reported for the quaternary alkylammonium counterion-modified TEMPO-oxidized CNFs, where the moisture content of the modified nanopaper was 7.0 wt % as compared with 10.5 wt % for the unmodified nanopaper at RH 50%.^24^ However, the mechanical properties of these modified nanopapers prepared from aqueous suspensions were compromised due to enhanced nanopore formation and decreased density of the dry nanocellulose film.^24,55^

**Figure 5:**
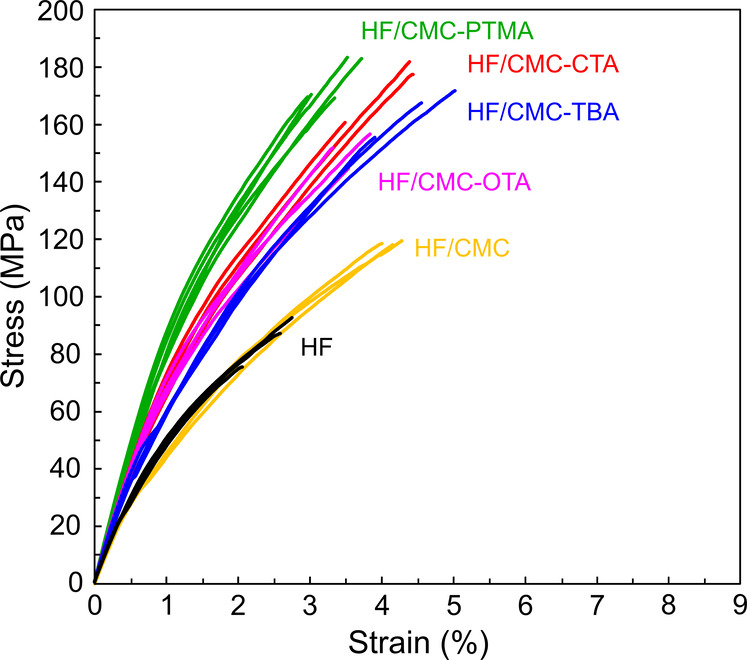
Typical tensile stress–strain curves of the HF/CMC-OTA, HF/CMC-TBA, HF/CMC-CTA, and HF/CMC-PTMA papers compared to the neat HF and HF/CMC papers at RH 50%.

The typical tensile stress–strain curves for the hydrophobic HF/CMC papers are shown in Figure 5 and the mechanical property data are summarized in Table S1. The addition of CMC did not increase the Young’s modulus of the HF papers but resulted in an increase in strain-to-failure from 2.5% to 4.2% as well as an increase in tensile strength from 84 to 117 MPa owing to improved fibril slippage. After functionalization with the quaternary ammonium counterions, the mechanical properties of the HF/CMC papers were enhanced significantly. The increased Young’s modulus (from 6.8 GPa to 7.9–9.4 GPa) and the 2-fold increased yield strength (from 45 to 84–95 MPa) suggested stronger fiber–fiber bonding in the HF/CMC papers functionalized with the QAs. The work to fracture was also improved from 1.3 to 3.3–4.5 MJ/m^3^ (Table S1). The work to fracture (toughness) was calculated from the area under tensile stress–strain curve, indicating the ability of the material to dissipate local high stress by enduring deformation.^29,56^ The synergy was achieved by increased density and reduced moisture in the fiber network as well as an enhanced fibril slippage through hydrophobic interactions. Such a synergistic effect indicated that modification on a wet HF paper structure could be beneficial, especially when the modification might alter the colloidal properties of the cellulosic fibers in the aqueous suspensions. The formation of a wet paper structure ensured a good fiber network; subsequent modifications on the preformed fiber network were feasible for a vast range of applications. The hydrophobic CMC was further applied as the compatibilizer at the interface in the HF/acrylic resin composites, as advanced fiber/polymer composites require good dispersion/distribution of the fiber network in the polymer matrix as well as strong interface strength between the fibers and polymer matrix.^4,16^

### 3.2 HF/ABPE Composites with Hydrophobic CMCs at the Interface

**Figure 6:**
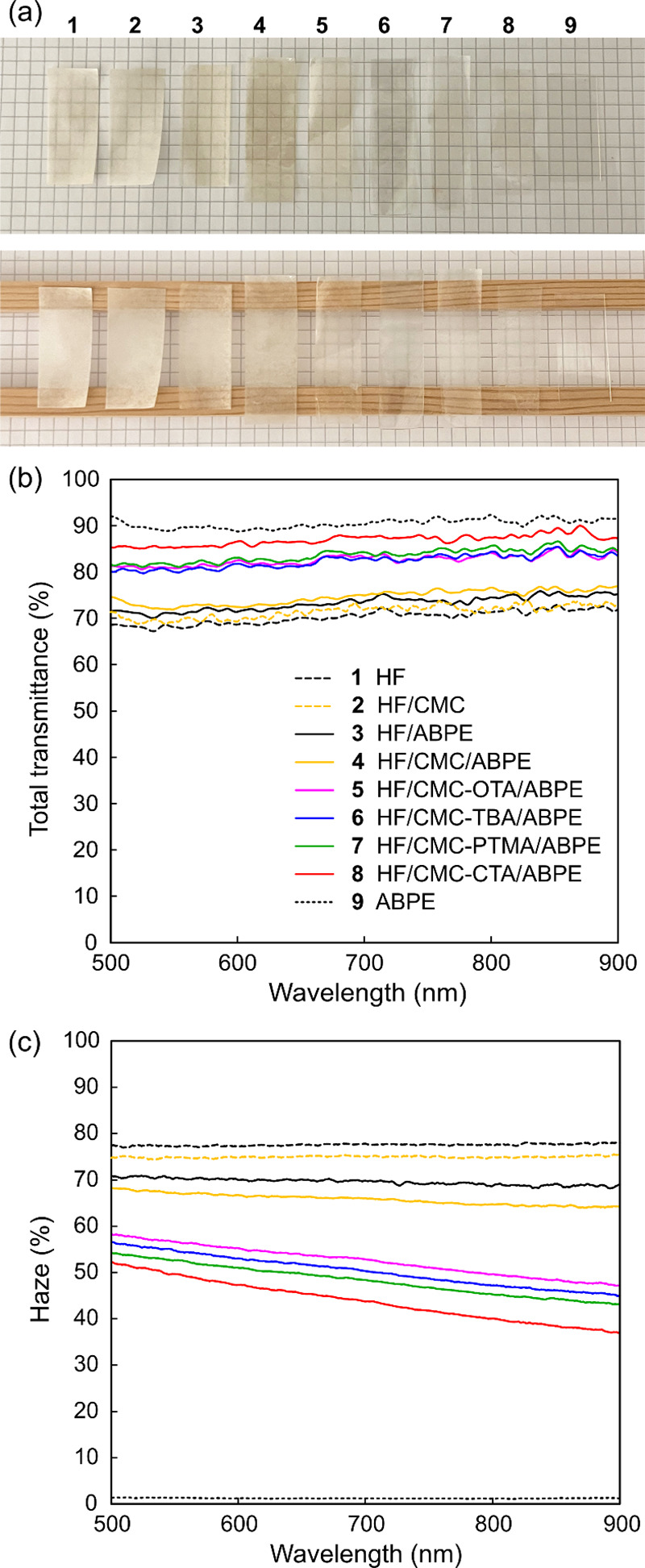
(a) Photograph of the HF/ABPE composite samples with and without CMC and hydrophobic functionalized CMCs in comparison with the neat HF, ABPE, and HF/CMC samples when placed on and 1 cm above a paper with grids. (b) Total transmittance and (c) haze of these samples in the wavelength range of 500–900 nm.

The hydrophobic HF/CMC papers were dried and impregnated with acrylic resin monomer and finally cured by UV light to obtain the HF/CMC-QA/ABPE composites with a controlled fiber content (fiber/resin = 60/40, w/w). The detailed compositions of the samples are summarized in Table 1. The effect of introducing CMC-QAs at the interface can be observed directly from the appearance of the composite films, as shown in Figure 6a. The HF/ABPE and HF/CMC/ABPE composites remained hazy after impregnation of acrylic resin, while the HF/CMC-CTA/ABPE film was significantly more transparent. The total transmittance and haze of the HF/ABPE composites with and without CMC and hydrophobic functionalized CMCs were measured using an integrating sphere. The film samples had a thickness of 60 μm, and the results are shown in Figure 6b,c. The neat ABPE film showed a total transmittance of 91%, with a haze of only 1.2%. The neat HF paper showed a high haze of 78% and a total transmittance of 70% due to the high porosity from the network composed of micron-size fibers. The introduction of CMC and further hydrophobic functionalization with the QAs resulted in a 4% increase in total transmittance and 5% decrease in haze for the HF/CMC-QAs papers (Figure S4), which was attributed to the CMC binding and filling the voids between the HF fibers as observed in the FE-SEM analysis (Figure 4 and Figure S3).

**Table 1:** Compositions of HF, ABPE, CMC, and QA as well as the Density and Porosity of the HF/ABPE Composites with Hydrophobic CMCs Functionalized with Different QAs

samples	HF	ABPE	CMC	QA	density (g/cm^3^)	porosity^a^ (%)
HF/ABPE	60.0	40.0			1.10	19.3
HF/CMC/ABPE	55.9	37.1	7.0		1.19	13.6
HF/CMC-OTA/ABPE	54.0	35.9	6.8	3.3	1.27	8.1
HF/CMC-TBA/ABPE	53.3	35.4	6.7	4.6	1.29	6.7
HF/CMC-PTMA/ABPE	54.2	36.0	6.8	3.0	1.30	5.9
HF/CMC-CTA/ABPE	52.9	35.1	6.6	5.4	1.30	6.1
ABPE		100			1.20	

a Porosity was calculated from apparent density and theoretical density, using ρ_HF_ and ρ_QA_ = 1.5 g/cm^3^, ρ_CMC_ = 1.6 g/cm^3^, and ρ_ABPE_ = 1.2 g/cm^3^.

Interestingly, with the incorporation of ABPE acrylic resin matrix in the HF paper, the total transmittance only improved from 70% to 73% (Figure 6b) while the haze reduced from 78% to 70% (Figure 6c), suggesting strong light scattering at the interfaces between acrylic resin and the unmodified HF fiber network. As the refractive index of ABPE10 is 1.516, the refractive index mismatching between fiber and polymer matrix could be ruled out. The high haze came from air voids and gaps on the undesirable interface in the composite material formed during impregnation, which led to light scattering instead of ballistic transmission of incident beam. By the introduction of hydrophobic functionalized CMC at the interface in the composites, the total transmittance increased, and the haze reduced more drastically for all the composites. A remarkable increase in total transmittance from 73% to 83–87% was recorded. This is comparable with the 78% to 88% increase in total transmittance reported for the covalent surface acetylation on BC to enhance the optical transparency of BC/TCDDMA (tricyclodecane dimethanol dimethacrylate) composites (thickness = 100 μm, fiber content 63 wt %).^5^ The highest optical transparency in this study was achieved by the composite with HF/CMC functionalized with CTAB, showing a total transmittance of 87% and reduced haze from 70% to 43%. This was consistent with the highest hydrophobicity observed for the HF/CMC-CTA paper, which led to improved impregnation with the hydrophobic acrylic resin, as further revealed by FE-SEM analysis.

**Figure 7:**
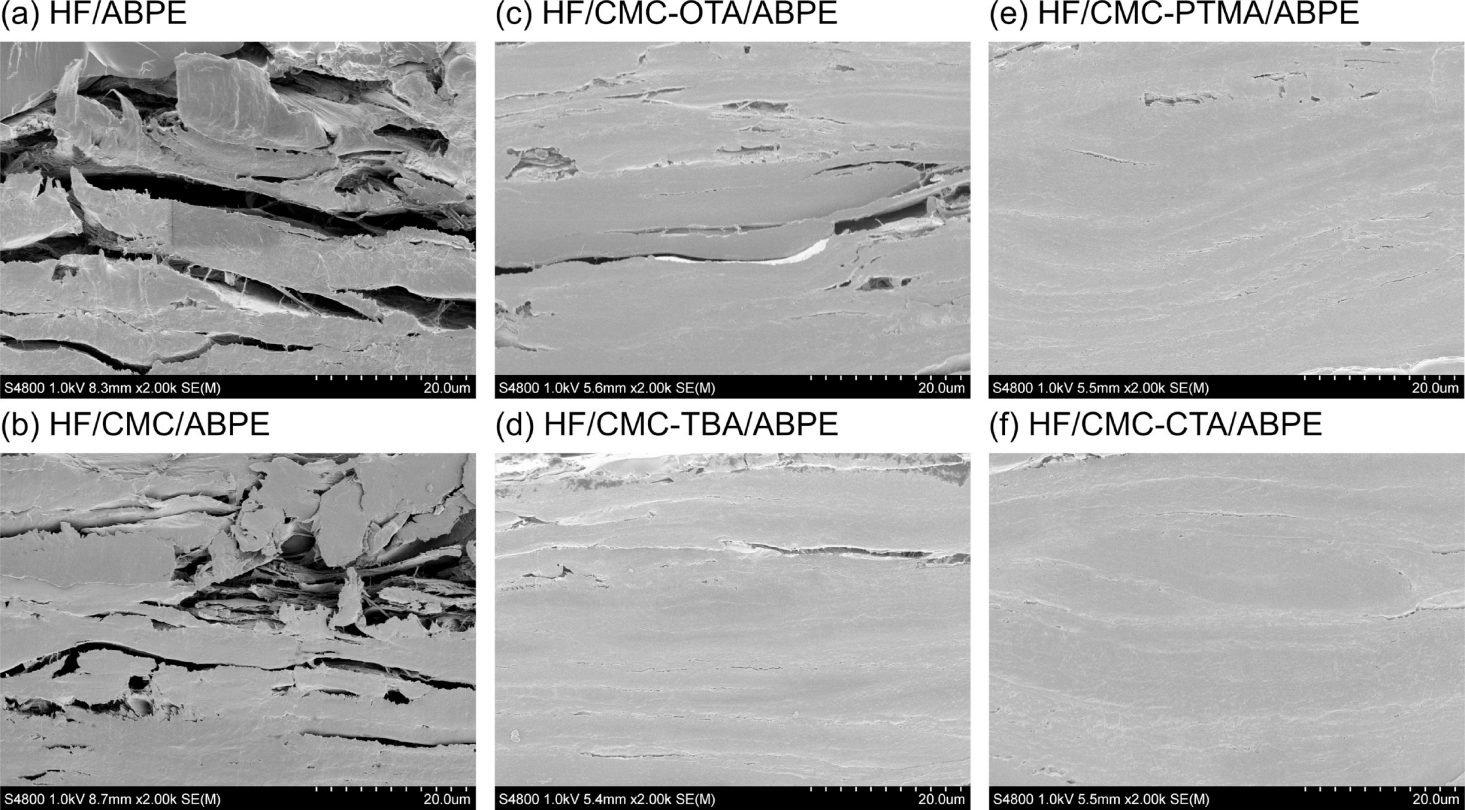
FE-SEM images of freeze-fractured cross sections of the HF/ABPE composites with and without CMC and hydrophobic functionalized CMCs.

To study the structure of these fiber–polymer composites, the samples were frozen in liquid nitrogen, and the fractured cross sections were imaged by FE-SEM, as shown in Figure 7. The large voids between fiber bundles and the resin matrix can be clearly seen in the HF/ABPE composite (Figure 7a). The infiltration of ABPE resin was inadequate, as the large gaps between fiber layers were not filled. These defects would not only cause light scattering leading to low transmittance and high haze but also serve as mechanical defects. With the presence of CMC-OTA at the interface, the infiltration was improved; however, small gaps between the fiber layers can still be observed (Figure 7c). This observation was consistent with relatively higher haze of the HF/CMC-OTA/ABPE composite as compared to composites with CMC bearing other QAs. A rather homogeneous impregnation of ABPE resin into fiber network was found in the HF/CMC-CTA/ABPE composite (Figure 7f), which explained its highest transparency and lowest haze. Comparing HF/ABPE composites with CMC-CTA and CMC-OTA at the interface, it is considered that a shorter alkyl chain (C-8 from OTA) as linear hydrophobic moieties is not adequate under this condition for a homogeneous infiltration of resin into fiber network, while longer alkyl chain (C-16 from CTA) indeed provided a much improved fiber–polymer interface due to higher hydrophobicity.

**Figure 8:**
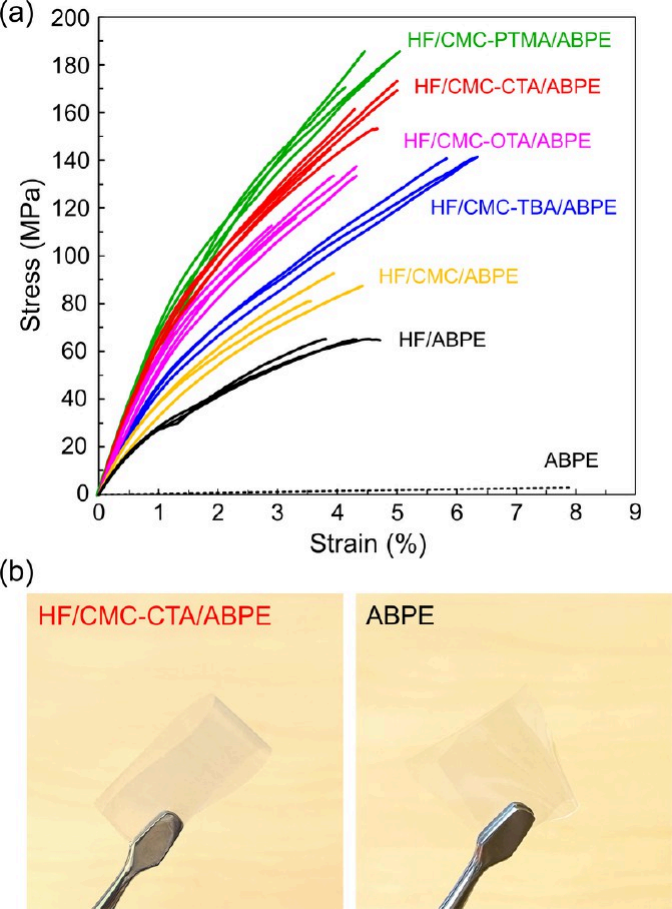
(a) Typical tensile stress–strain curves of HF, HF/ABPE composites with and without CMC, and hydrophobic functionalized CMCs at 50% RH. (b) Transparent and foldable HF/CMC-CTA/ABPE composite and fragile neat ABPE sheet of the same thickness (60 μm).

The effect of hydrophobic functionalized CMC at the interface on mechanical performance of the HF/ABPE composites were investigated by tensile tests and the typical stress–strain curves are shown in Figure 8a. Their mechanical property data are summarized in Table S2. Neat ABPE was a relatively weak polymer, with a tensile strength of 3.1 MPa and a strain to failure of 8.1%. The neat ABPE resin sheet was fragile to handle, and breakage easily occurred when the resin sheet was folded with a tweezer (Figure 8b). The HF/ABPE composite containing 60 wt % HF showed a Young’s modulus of 3.8 GPa, yield strength of 24 MPa, and tensile strength of 63 MPa, significantly lower than those for the neat HF paper (Figure 5). Such low mechanical properties resulted from the debonding of the fiber–polymer matrix interface as revealed from the freeze-fractured cross-sectional FE-SEM images (Figure 7a). With the introduction of hydrophobic functionalized CMC at the interface in the HF/ABPE composites, their mechanical performances were significantly enhanced. The work-to-fracture (toughness) values were 4.5, 5.2, and 5.2 MJ/m^3^ for the composite containing CMC functionalized with CTA, TBA, and PTMA quaternary ammonium ions (Table S2), respectively. These values were 2 times higher than that of the HF/ABPE composite without interface modifications (1.7 MJ/m^3^). Particularly, CMC-PTMA bearing a phenolic ring resulted in the HF/CMC-PTMA/ABPE composite with the highest Young’s modulus of 7.6 GPa, yield strength of 83 MPa, and tensile strength of 180 MPa followed by CTA with a C-16 alkyl chain, which resulted in comparable Young’s modulus of 7.4 GPa, yield strength of 81 MPa, and tensile strength of 159 MPa. On the other hand, CMC-TBA with 4 branched C4 alkyl chains resulted in the highest strain to failure of 6.1% in the tensile deformation for the HF/CMC-TBA/ABPE composite. These composites were all foldable as represented by the HF/CMC-CTA/ABPE composite film in Figure 8b.

Transparent, thin, and flexible composite films from cellulosic fibers and polymer resins are favorable for optoelectronic applications due to low thermal expansion coefficient, lightweight and high strength.^39^ These composites have been prepared using nanocellulose including BC and CNFs, combined with covalent modifications, such as acetylation.^5,36,37,57,58^ If such composites can be produced from HF instead of cellulose nanofibers, then the energy-extensive process required by the production of BC and CNFs can be avoided. HF paper contains pores at both macro- and nanoscales, which induce light scattering and result in low transmittance and high haze. Therefore, the infiltration of polymer resin and the fiber–polymer interfaces must be tailored in order to achieve high optical transparency as well as good mechanical performances. Instead of the covalent modification method, the hydrophobic CMC bearing quaternary ammonium counterions successfully resolved this challenge and resulted in strong and transparent HF-based composites compared to those based on cellulose nanofibers in the literature as summarized in Table 2. The HF/CMC-CTA/ABPE10 and HF/CMC-PTMA/ABPE10 composites from this work showed the highest Young’s modulus and tensile strength as well as comparable optical transmittance at a fiber content over 50 wt %.

**Table 2:** Comparison of Cellulose Content, Young’s Modulus, Tensile Strength, Total Transmittance (*T*), and Thickness of Various Cellulose/ABPE Composites from the Literature^a^

composites	cellulose content (wt %)	Young’s modulus (GPa)	tensile strength (MPa)	*T* (%)	thickness (μm)	ref
CNF/ABPE	66	5.2 (1.4)	175.1 (5.1)	85.7	26.1^b^	57
CNF/ABPE	26	2.1 (0.2)	32.3 (2.3)	87.9	100	58
CNF/ABPE	25	1.7 (0.2)	41 (2.6)	89.4	100	58
acetylated CNF/ABPE	68	4.06	173.7	82.5	45	37
acetylated CNF/ABPE	35–40	3–4	90	82–85	90–100	36
BC/ABPE	5	0.355	20	81.3	700	39
acetylated BC/ABPE	63	-	-	87.8	100	5
HF/CMC-PTMA/ABPE	54.2	7.6 (0.5)	180 (7)	84	60	this work
HF/CMC-CTA/ABPE	52.9	7.4 (0.3)	159 (10)	87	60	this work

a The values in parentheses are the sample standard deviations.

b Before impregnation.

## 4 Conclusions

The unfavorable impregnation of acrylic resin into the hydrophilic holocellulose fiber network caused the formation of defects at the interfaces, compromising both the mechanical robustness and optical transparency of the composite. We demonstrated a facile interface tailoring method alternative to covalent surface modification on holocellulose fibers without the use of organic solvents and harsh chemicals. The introduction of a CMC that was hydrophobic functionalized by quaternary ammonium counterions at the fiber/polymer interface significantly enhanced the optical transparency and mechanical performances of the HF/acrylic resin composites. The chemical structure of the QA ions has a strong effect on the property enhancement of the composites. Functionalization with QAs bearing a long alkyl chain (CTA) or a phenolic ring (PTMA) demonstrated high hydrophobicity, improved infiltration of acrylic resin and interfacial adhesion, high optical transparency and low haze, and excellent mechanical performance. Utilizing hydrophobic quaternary ammonium-functionalized carboxymethyl cellulose as the interface compatibilizer enhances impregnation/infiltration of hydrophobic polymer in cellulosic fiber network and improves interfacial adhesion between the fiber and polymer. This method offers innovative avenues for designing cellulosic fiber/polymer composites with improved optical and mechanical performances in an energy-efficient and environmentally friendly manner.

## Supporting Information Available

FE-SEM image of spruce holocellulose fibers; water contact angles of HF/CMC papers treated with different amounts of CTAB; FE-SEM surface images, total transmittance, and haze of the HF/CMC-QAs papers; mechanical property data of the HF/CMC-QAs papers and the HF/CMC-QAs/ABPE composites films (Table) (PDF)
