# Supplementary material for: Tailoring the Holocellulose Fiber/Acrylic Resin Composite Interface with Hydrophobic Carboxymethyl Cellulose to Enhance Optical and Mechanical Properties
Source: Biomacromolecules. 2024 May 7;25(6):3731–40. doi: 10.1021/acs.biomac.4c00295 (PMC11170952; doi:10.1021/acs.biomac.4c00295)
Supplement: Supplementary file 1 — bm4c00295_si_001.pdf [file bm4c00295_si_001.pdf]

Supporting Information

Tailoring the Holocellulose Fiber/Acrylic Resin Composite Interface with Hydrophobic Carboxymethyl Cellulose to Enhance Optical and Mechanical Properties

*Li Zha [a], Max Yan[b], Lars A. Berglund[c], Qi Zhou [a,c]**

[a] Division of Glycoscience, Department of Chemistry, School of Engineering Sciences in Chemistry, Biotechnology and Health, KTH Royal Institute of Technology, AlbaNova University Centre, SE-106 91 Stockholm, Sweden

[b] Department of Applied Physics, School of Engineering Sciences, KTH Royal Institute of Technology, SE-114 19 Stockholm, Sweden

[c] Wallenberg Wood Science Center, Department of Fibre and Polymer Technology, KTH Royal Institute of Technology, Teknikringen 56, SE-100 44 Stockholm, Sweden

* Corresponding author. Tel: +46 8 790 96 25, e-mail: qi@kth.se

This PDF file of Supporting Information includes:

Supplementary Figures S1 to S4

Supplementary Tables S1 to S2

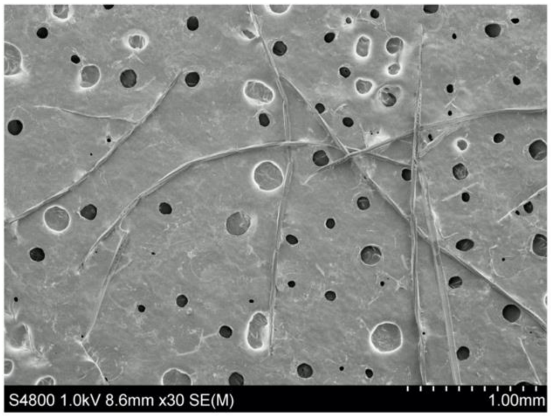

Figure S1. SEM image of spruce holocellulose fibers.

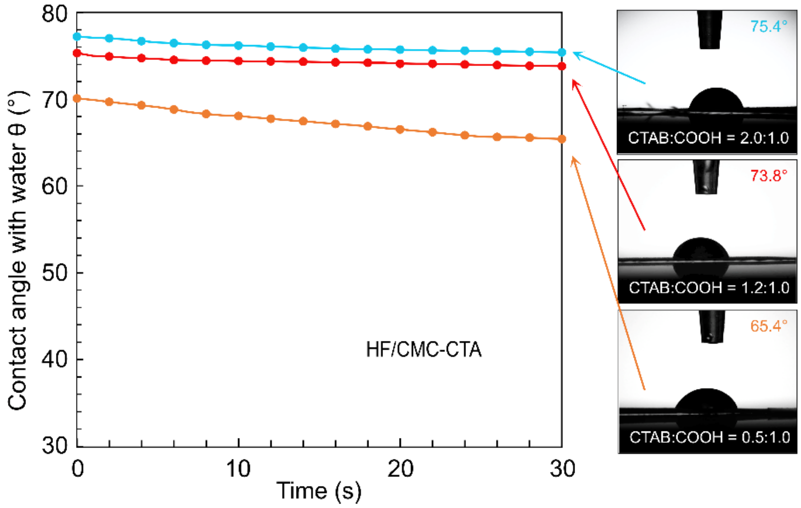

Figure S2. Time dependent contact angles of water droplets on the HF/CMC papers treated with different amount of CTAB.

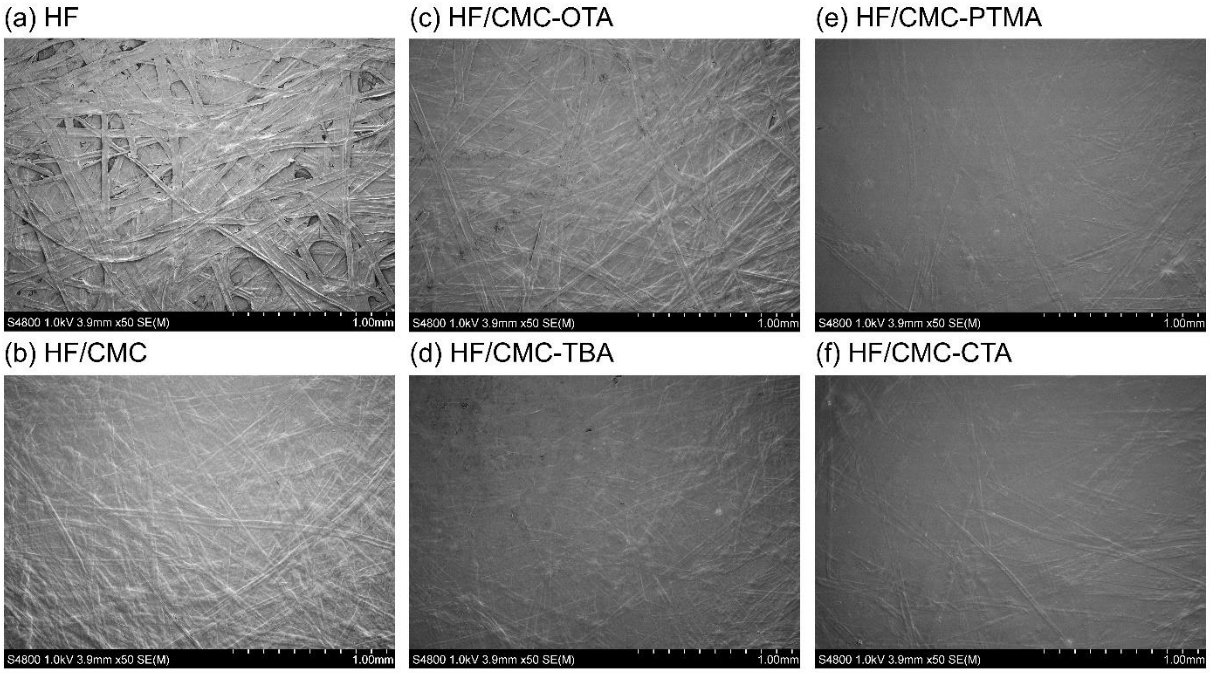

Figure S3. FE-SEM surface images of the HF/CMC-OTA, HF/CMC-TBA, HF/CMC-PTMA, and HF/CMC-CTA papers as compared with the neat HF and HF/CMC papers.

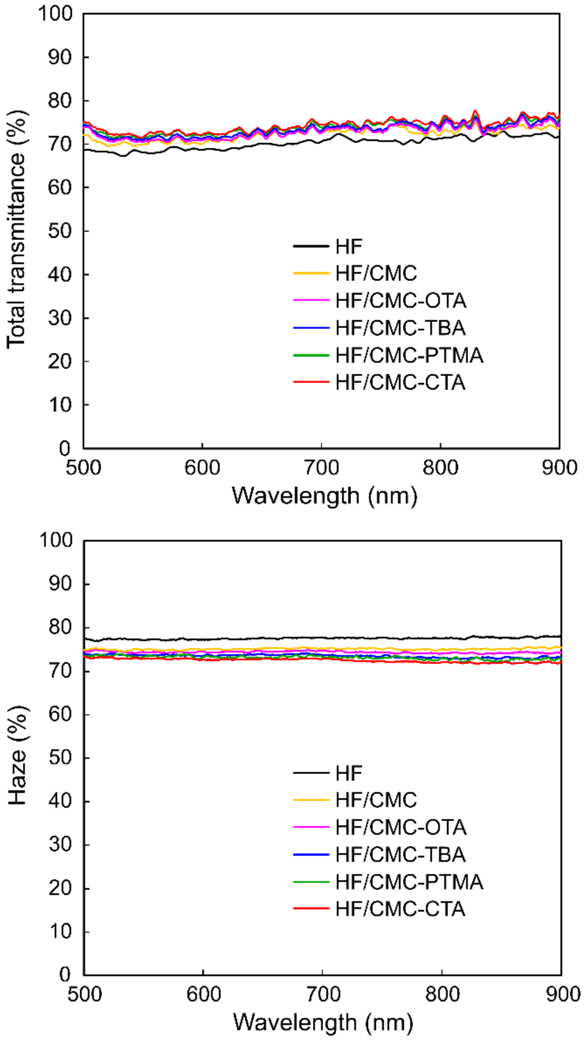

Figure S4. Total transmittance and haze of the HF papers with and without hydrophobic functionalized CMC in the wavelength range of 500–900 nm.

Table S1. Physical and mechanical properties data of the HF/CMC-OTA, HF/CMC-TBA, HF/CMC-CTA, and HF/CMC-PTMA papers as compared with the neat HF and HF/CMC papers.[a]

Holopapers	Tensile strength (MPa)	Strain to failure (%)	Young's Modulus (GPa)	Yield strength (MPa)	Work to fracture (MJ/m^3)	Density (g/cm^3)	Moisture Content (wt.%)
HF	84 (9)	2.5 (0.4)	6.8 (0.4)	45 (3)	1.3 (0.3)	0.75	11.3
HF/CMC	117 (5)	4.2 (0.2)	6.7 (0.3)	40 (4)	3.0 (0.2)	0.82	12.5
HF/CMC-OTA	151(6)	3.6 (0.3)	8.6 (0.5)	88 (4)	3.3 (0.4)	0.86	7.0
HF/CMC-TBA	165 (9)	4.5 (0.6)	7.9 (0.4)	84 (4)	4.5 (0.9)	0.85	7.1
HF/CMC-PTMA	174 (8)	3.3 (0.3)	9.4 (0.6)	95 (3)	3.5 (0.5)	0.89	6.4
HF/CMC-CTA	172 (9)	4.1 (0.4)	8.7 (0.3)	93(3)	4.3 (0.6)	0.91	6.3

[a] The values in parentheses are the sample standard deviations.

Table S2. Mechanical properties data, total transmittance (*T*) and haze of HF/ABPE composites with and without CMC and hydrophobic functionalized CMCs at RH 50%.[a]

Samples	Tensile Strength (MPa)	Strain to failure (%)	Modulus (GPa)	Yield strength (MPa)	Work to fracture (MJ/m^3)	*T*% (%)	Haze (%)
HF/ABPE	63 (3)	4.3 (0.5)	3.8 (0.2)	24 (4)	1.7 (0.3)	73	70
HF/CMC/ABPE	87 (6)	4.0 (0.4)	4.5 (0.5)	35 (4)	2.1 (0.3)	75	66
HF/CMC-OTA/ABPE	126 (12)	3.8 (0.6)	6.6 (0.5)	63 (5)	3.1 (0.9)	83	52
HF/CMC-TBA/ABPE	140 (4)	6.1 (0.3)	5.4 (0.2)	47 (6)	5.2 (0.3)	83	50
HF/CMC-PTMA/ABPE	180 (7)	4.6 (0.4)	7.6 (0.5)	83 (4)	5.2 (0.8)	84	48
HF/CMC-CTA/ABPE	159 (10)	4.2 (0.5)	7.4 (0.3)	81 (4)	4.5 (0.7)	87	43
ABPE	3.1 (0.2)	8.1 (1.0)	0.04 (0.01)	-	0.13 (0.01)	91	1.2

[a] The values in parentheses are the sample standard deviations.
